# The Acceptability of a Real-Time Medication Monitoring-Based Digital Adherence Tool Among Young People Living with HIV in Malawi

**DOI:** 10.1007/s10461-025-04885-7

**Published:** 2025-09-23

**Authors:** Takondwa Charles Msosa, Felix Phuka, Marion Sumari-de Boer, Owen Mhango, Hussein Hassan Twabi, Madalo Mukoka, Iraseni Swai, Rob Aarnoutse, Tobias F. Rinke de Wit, Kennedy Ngowi, Edred Lunda, Wongani Mphande, Chisomo Msefula, Marriott Nliwasa

**Affiliations:** 1 Amsterdam UMC, Department of Global Health, location University of Amsterdam, Amsterdam, The Netherlands; 2 Amsterdam Institute for Global Health and Development, Amsterdam, The Netherlands; 3 Helse Nord Tuberculosis Initiative, Department of Pathology, Kamuzu University of Health Sciences, Blantyre, Malawi; 4 Kilimanjaro Clinical Research Institute, Moshi, Tanzania; 5 Kilimanjaro Christian Medical College University, Moshi, Tanzania; 6 Department of Pharmacy, Pharmacology and Toxicology, Radboud University Medical Center, Research Institute for Medical Innovation, Nijmegen, The Netherlands; 7 Institute of Life Course and Medical Sciences, University of Liverpool, Liverpool, UK

**Keywords:** HIV, Antiretroviral therapy, Young people, Digital adherence tools, Real-time medication monitoring, Acceptability

## Abstract

Adherence to antiretroviral therapy (ART) is required for young people living with HIV (YPLHIV, 15-24 years) to achieve viral suppression, prevent transmission, and reduce HIV-related morbidity and mortality. Real time medication monitoring based digital adherence tools (RTMM-DATs) - a combination of RTMMs (smart pillboxes), customised adherence feedback, SMS reminders, and optional alarms - may be used to provide promising interventions to improve ART adherence and viral suppression in YPLHIV. In this study, we sought to investigate the concurrent acceptability of a RTMM-DAT intervention which included RTMMs, customised adherence feedback, SMS reminders, and optional alarms, in non-adherent YPLHIV on ART in Malawi enrolled in a randomised controlled trial (RCT) to investigate the effect of the intervention on ART adherence and viral suppression. As a secondary objective, we explored barriers to ART adherence in YPLHIV using the intervention. We conducted a phenomenological study in fourteen YPLHIV enrolled in the intervention arm of the RCT for at least six months using semi-structured interviews between June and July 2024. The interview guide was structured around the constructs of the Theoretical Framework of Acceptability (TFA). Inductive and deductive thematic analysis were performed to achieve the objectives. Overall, the participants expressed positive attitudes toward the intervention and appreciated the portable and discreet design of the RTMM. They appreciated the intervention's usefulness in addressing forgetfulness and promoting routine medication use. Neutral SMS reminders and tailored adherence feedback were appreciated for promoting adherence while safeguarding privacy. Challenges included device recharging in settings with limited electricity, reliance on mobile phones for SMS reminders, lack of remote user access to adherence reports, potential unintended disclosure from audible alarms, and users’ underlying social insecurities due to stigma. Furthermore, participants continued to face multiple and complex challenges to ART adherence, such as food insecurity, drug side effects, forgetfulness, and social stigma before and after engaging with the intervention. In conclusion, the intervention was generally acceptable among YPLHIV in Malawi. Addressing barriers such as electricity access, stigma, and enhancing user customisability and access to adherence reports could improve the intervention’s acceptability. Additionally, tailoring the intervention to the socio-cultural context of YPLHIV is essential to improve acceptability.

**Supplementary Information:**

The online version contains supplementary material available at 10.1007/s10461-025-04885-7.

## Background

Adherence to antiretroviral therapy (ART) is crucial for young people living with HIV (YPLHIV, 15–24 years) [1, 2], who often face unique challenges in maintaining consistent medication intake [3, 4]. ART adherence in this demographic is adversely affected by a variety of factors including stigma, ART side effects, mental health issues, concerns about privacy, and the complex dynamics of transitioning from childhood to adulthood with the addition of an HIV diagnosis [5–8]. Poor adherence to ART can lead to virologic failure [9–11], drug resistance [12–14], and increased risk of HIV transmission [15–18], making adherence support strategies and interventions critical for improving treatment outcomes in this population and reducing HIV-associated morbidity and mortality.

In the past 20 years, digital adherence tools (DATs) have been used to provide promising interventions to support ART adherence in PLHIV [19–21]. DATs such as real time medication monitors (RTMMs), which are pillboxes that record in real-time the opening of the box, coupled with SMS reminders and customised adherence feedback, offer real-time monitoring and personalised feedback to enhance ART adherence in PLHIV [21, 22]. While there is evidence on the efficacy and acceptability of RTMM-based DATs (RTMM-DATs) in adult PLHIV [21–27], data on the efficacy and acceptability of these interventions in YPLHIV in sub-Saharan Africa is limited [22]. Evidence from China has shown that real time electronic drug monitoring is generally acceptable in adolescents living with HIV [28], however, these findings cannot be generalised to African settings like Malawi due to significant sociocultural and economic differences.

Young people are often early adopters of technology and may be more receptive to digital health interventions [29]. However, factors such as user friendliness, privacy concerns, and the socio-cultural context in which digital health interventions are implemented can significantly influence the acceptability of digital health interventions and subsequently their effectiveness [30]. Therefore, understanding how YPLHIV perceive and interact with RTMM-DATs is essential for designing interventions that are both effective and acceptable in this demographic.

The primary objective of this study was to explore the concurrent acceptability of a RTMM-DAT among YPLHIV enrolled in a randomised controlled trial investigating the effect of RTMM-DATs on ART adherence and viral suppression in YPLHIV in Malawi [31].

The secondary objective was to explore challenges to ART adherence that YPLHIV faced before and after being enrolled into the study.

The intervention targeted YPLHIV who were on ART but had suboptimal adherence (< 80%) based on clinic pill counts. The intervention had 3 main components: a RTMM, SMS reminders, and personalised adherence feedback. The alarm was an optional feature. The RTMM pillbox, developed by Wisepill™, transmitted pillbox opening data via to a secure server. Participants received SMS reminders before and after scheduled dosing times if no opening was detected. The messages were neutral to protect confidentiality. Adherence feedback was generated from the server data and used for counselling during clinic visits.

At the time of the qualitative interviews, the study had recruited a total of 120 participants in each arm respectively and participants were undergoing follow-ups. More information on the trial design has been described in detail elsewhere [31].

## Methods

### Research Team

Data collection, coding, and analysis were primarily conducted by two investigators (TCM and FBP), both trained in qualitative research methods and fluent in Chichewa and English. TCM has a medical background, while FBP is a social scientist. No prior relationships existed between the researchers and participants before the study. To minimise researcher bias, both researchers engaged in reflexivity throughout data collection and analysis by maintaining reflective journals.

### Study Design

We employed a descriptive phenomenological design, an approach that seeks to explore and understand how individuals experience a particular phenomenon, focusing on their own lived experiences and perceptions, and not of those of the researcher. This approach allowed us to explore the lived experiences of YPLHIV actively using the intervention and to understand barriers to ART adherence from their perspective.

The Theoretical Framework of Acceptability (TFA) was used as the guiding framework to assess participants’ perceptions and experiences regarding the intervention [30]. The TFA is a comprehensive model, made up of seven constructs, that is used to assess the acceptability of the intervention for its recipients and implementers. The constructs of the TFA are described in detail in Table 1.

**Table 1:** Description of the domains of the TFA

Construct	Definition
Affective Attitude	How an individual feels about the intervention
Burden	The perceived amount of effort required to participate in the intervention.
Ethicality	The extent to which the intervention aligns with an individual’s values and ethical beliefs.
Intervention Coherence	The extent to which an individual understands the intervention and how it works.
Opportunity Costs	The extent to which benefits of the intervention outweigh other opportunities or alternatives.
Perceived Effectiveness	The extent to which an individual perceives the intervention as likely to achieve its purpose.
Self-efficacy	The individual’s confidence in their ability to engage with and complete the intervention.

The TFA was selected because it directly addressed the focus of our research, which was to explore the subjective experience of participants using the intervention and how their experience influenced its acceptability. Furthermore, unlike other frameworks, the TFA specifically theorises acceptability as a multifaceted construct influenced by anticipated and experienced cognitive and emotional responses, making it particularly relevant for assessing the acceptability of complex interventions like the RTMM-DAT in YPLHIV [30]. Additionally, the TFA has been implemented successfully in similar contexts when assessing the acceptability of DATs in PLHIV and people with other medical conditions [23, 32, 33].

### Study Population and Recruitment

We interviewed a total of 14 participants who were enrolled in the intervention for at least 6 months. Participants were selected purposively to ensure balance in age and sex. Data saturation was reached by the 12th interview, however, we completed all 14 interviews as initially planned. The point of data saturation was consistent with empirical data in the field which shows that 6 to 20 interviews are enough to reach saturation when describing a narrow phenomenon from a homogenous group of participants, which was the case in our study [34–36]. No person who was approached by the study team refused to participate in the interviews.

Recruitment was facilitated by two research assistants (EL and WM) at the ART clinics of Limbe and Ndirande Health Centres respectively. The research assistants introduced the study to the participants via telephone, and those who were willing to participate were invited to the ART clinics where they were approached by one of the two investigators. Information sheets were then provided to all would-be participants. Written informed consent was obtained from adult participants and the legal guardians of participants who were minors. Additionally, participants who were minors were asked to provide assent before recruitment into the study.

### Data Collection

Semi-structured in-depth interviews were conducted with each participant between June and July 2024 using an interview guide based on the seven constructs of the TFA [30]. The interview guide had three main sections: the participants’ sociodemographic and contextual background, their experiences with HIV care and treatment, and their perceptions and attitudes toward the digital adherence intervention based on the TFA constructs. The interview guide can be seen in Supplemental Material 1.

The interview guide was piloted to ensure accuracy, clarity and depth. The interviews took place face-to-face in a private room at the HIV care and treatment clinics of the study sites, ensuring a comfortable and confidential environment for participants. Interviews were conducted in Chichewa, the dominant local language, by two of the investigators (TCM and FBP). Each interview lasted approximately 60 min and was audio-recorded with the participants’ consent. In addition to the recordings, field notes were taken during each interview to capture non-verbal cues, contextual details, and the interviewers’ reflections and observations.

### Data Analysis

The audio-recorded interviews were translated and transcribed verbatim directly to English by two investigators (TCM and FBP). Participants were not given a copy of their transcripts to review due to psychological considerations given the sensitivity of the topic of discussion. We employed a combination of inductive and deductive thematic analyses guided by the TFA domains. Audio recordings and transcripts were listened to and read multiple times to ensure immersion in the data.

Two independent researchers (TCM and FBP) coded the transcripts to ensure inter-coder reliability. A codebook was generated using deductive and inductive coding frameworks. Coding discrepancies were resolved through discussion and consensus by TCM and FBP. NVivo (version 14, QSR International) was used for coding and for data management and organisation.

### Ethical Considerations

The study was approved by the College of Medicine Research and Ethics Committee (COMREC) (approval number: P.12/23–0494). All participants provided written informed consent, and for participants under 18 years of age, apart from consent of their parents, written assent was also obtained. To ensure confidentiality, all identifying information was anonymised during data collection, analysis, and reporting. Participants were informed about their right to withdraw from the study at any time without repercussions.

### Description of the Intervention

The intervention enrolled YPLHIV who were on ART but not adherent to their medication: adherence of less than 80% based on pill counts at their respective primary health facilities. The intervention (RTMM-DAT) had three main components: a RTMM (smart pillbox), reminder SMSs, and customised adherence feedback. The alarm functionality of the RTMM was optional. The RTMM is a smart pillbox developed by Wisepill™ containing a SIM card that sends data to a central server through the worldwide general packet radio service (GPRS) network. The pillbox can hold approximately 30 large pills or 60 small pills [37].

Data about opening events from the pillbox are sent to and stored on a secure server. The data includes information about the opening time, the pillbox’s identification number, and battery and signal strength specifications. The pillboxes were linked to mobile phones from which participants received reminder SMSs 30 min before their medication intake time and triggered reminders one hour later should an opening event not be recorded on the server.

To maintain confidentiality and unintended disclosure, the SMS reminders were neutral and unrelated to their HIV status. The customised adherence feedback was based on medication adherence reports stored on the server and accessed by the research team. At each clinic visit, participants were given these reports and counselled based on their performance according to the reports. More information on the intervention design and theory of behavioural change have been published elsewhere [27, 31, 32].

## Results

A total of fourteen participants were included in the analysis. The age range of participants was between 15 and 24 years of age. 8 participants were female and 6 were male. Most of the participants (11 out of 14) acquired HIV vertically according to their medical history. In terms of education, 10 were in secondary school, 3 in primary school, and 1 was undertaking tertiary education. Participant details can be seen in Table 1.

In the thematic analysis, in addition to the 7 domains of the TFA, one additional theme was generated, adherence to ART, which described participants’ experiences and barriers to ART adherence before and after the introduction of the intervention. This was an important theme because it described the context in which the intervention was being implemented and the lived experience of young people managing their HIV diagnosis while navigating these complex challenges.

**Table 1:** Participants, demographic information

Identification	Sex	Age	Marital status	Current education	Probable route of Infection
Participant 1	Male	18	Single	Secondary school	Parent to Child Transmission
Participant 2	Male	22	Single	Secondary school	Parent to Child Transmission
Participant 3	Female	24	Separated	Primary school	Horizontal
Participant 4	Female	20	Separated	Primary school	Horizontal
Participant 5	Male	21	Single	Secondary school	Parent to Child Transmission
Participant 6	Female	22	Single	Tertiary	parent to Child Transmission
Participant 7	Male	18	Single	Secondary school	Parent to Child Transmission
Participant 8	Female	15	Single	Secondary school	Parent to Child Transmission
Participant 9	Male	17	Single	Secondary school	Parent to Child Transmission
Participant 10	Male	21	Single	Secondary school	parent to Child Transmission
Participant 11	Female	24	Single	Secondary school	Parent to Child Transmission
Participant 12	Female	16	Single	Secondary school	Parent to Child Transmission
Participant 13	Female	21	Single	Secondary school	Parent to Child Transmission
Participant 14	Female	23	Separated	Primary school	Horizontal

### Adherence to ART

Before and after being enrolled in the intervention, participants expressed various challenges to adhere to ART. Barriers to ART adherence that participants reported were food insecurity, drug side effects, forgetfulness, and social stigma, which collectively contributed to inconsistent medication adherence.

#### Food Insecurity

Food insecurity emerged as a significant challenge, with many participants expressing difficulty adhering to ART due to the occasional lack of food.

In the recent past, whenever I took medication on an empty stomach, I used to experience nausea. So, whenever that happened, I could just stop and not take the drugs until I found food to eat. (Participant 12)

The fear of experiencing side effects, or the worsening of side effects when taking medication on an empty stomach, was a prominent concern. This fear often led to intentional delays or outright avoidance of taking medication until food was available.

One other time I was afraid to take medication on an empty stomach fearing that I might just die or fall down suddenly. (Participant 10)

Another thing is that drugs make you feel nauseous if taken before eating. I could wait for food first, otherwise I couldn’t take drugs if I didn’t eat. (Participant 13)

#### Drug Side Effects

Participants expressed drug side effects as one of the major barriers to adherence which made them opt out of taking their medication consistently to avoid feeling sick. Participants expressed that side effects, particularly dizziness and nausea, made them reluctant to take their medication consistently.

It just happens that sometimes I could forget and sometimes after taking the medication, I’d experience dizziness, feeling as if I am sick. (Participant 11)

#### Forgetfulness

Forgetfulness also emerged as an important theme in relation to barriers to adherence. Several participants reported that they occasionally forgot to take their medication due to various reasons.

In the past, I had difficulties remembering my schedule, I could miss my schedule many times. I could even miss and skip for several days. Maybe missing my dosage today and possibly remember to take it the next day. (Participant 9)

Forgetfulness was often linked to disruptions in their daily routines, such as being preoccupied with other activities or being away from home.

I could find myself forgetting. Maybe in a month I could forget once, thrice. Sometimes when I’ve gone to the market to buy things, maybe I’d come back a bit late, because at the market you encounter so many attractive things. Only to find that your schedule for taking medication has elapsed. (Participant 4)

Busy schedules due to competing priorities were also noted as barriers to adherence. Participants could miss their drug schedule due to their preoccupation with other equally important economic activities.

When I was engaged in business, it could be difficult since I could possibly knock off a bit late. Depending on the time I knocked off from the business, the schedule for taking drugs could not be consistent. (Participant 3)

#### Social Stigma

In terms of social stigma, some participants revealed that they would sometimes skip doses to avoid unintentionally disclosing their HIV status to others. The fear of being stigmatised within their community led participants to conceal their medication use, particularly when they were in the presence of family members or visitors who were unaware of their status.

Let’s say some relatives are visiting, some of my relatives don’t know that I have HIV. So, let’s say they’re visiting…, and you may probably be aware that some visitors may not wake up early and leave the bedroom. They may sometimes wake up very late, maybe around 10 am and sometimes they may stay in bed up to around 11 am. Sometimes I delay taking my medication until they move away……. When people know that someone is HIV positive in this country, they discriminate against them. (Participant 5)

The need for privacy or lack of private space among YPLHIV came out as a prominent determinant to adherence because in the instance where their privacy was threatened or compromised, participants were deterred from observing their ART intake schedules. This point came out from almost all participants expressing their discomfort to take medication in the presence of strangers or unfamiliar people.

The time I received a visitor, it was difficult for me to comply with the treatment. When I have failed to take treatment in the morning, I don’t take it in the afternoon because I have missed the prescribed time. (Participant 13)

I was not even complying with the treatment, one week could pass without taking the drugs because I was sleeping with my sisters and brothers in the same house. There were no rooms for each one of us, it was just one house for us, it was difficult for me to take drugs. (Participant 14)

### Acceptability of the RTMM DAT

#### Affective Attitude

The participants generally expressed a positive affective attitude toward the intervention and appreciated the intervention’s role in supporting consistent medication intake. The participants were positive about the design of the RTMM (pillbox) and the contents of the reminder SMSs.

The way the gadget is designed you can even carry it, it’s simple; you can carry it with you without people knowing that inside it you’re carrying medicine. It’s a well-protected and decent gadget. (Participant 4)

No, it makes me happy, I’m now used to it because I lacked something that could always remind me to keep time. I missed (taking medication) on time because I lacked something that could remind me. (Participant 10)

The SMS alerts, I like them because when the message comes…, the first message comes at half past five to alert you that you shouldn’t forget that it’s about time to take my medication. (Participant 11)

Although the alarm was an optional feature, participants who opted for it had mixed feelings about it and some requested that it should be disabled. While some appreciated its functionality and perceived it as a helpful tool in reminding them to take medication, others were concerned about the audible cue due to the risk of unwarranted attention or unintended disclosure.

As for me, the main thing concerns the alarm, especially in a strange place. I’ve no problem with the SMS because they’re not suspicious. (Participant 5)

In terms of the customised adherence feedback, participants were generally positive about this aspect of the intervention, expressing how the feedback motivates them to take their ART as prescribed.

The feedback is good that even as before you go to the clinic you already know that things will probably not be well with me or that my results will not be impressive. But if you’re consistent, you have nothing to fear because you already know that you haven’t messed up and you go there with courage. (Participant 10)

#### Perceived Burden

Even though most participants reported no significant burden with participating in the intervention, several participants expressed concerns about the burden associated with maintaining the RTMM. Although the device is rated to last for a maximum of six months on a full charge, some participants expressed challenges in recharging the device periodically, which proved difficult for those with limited or no access to electricity. Participants who did not have reliable electricity at home had to find alternative locations to recharge the device, which was inconvenient for them.

Because it’s obvious for all of us using that gadget that it can indicate whether someone is adherent or not, but sometimes it happens that maybe it has run out of power. For example, the way I’ve explained about my electricity challenge, I may take the drugs from the gadget…, will it be able to indicate in the server that I’ve dispensed the drugs considering that it’s switched off? I also think that could be some kind of challenge somehow. (Participant 5)

Despite this, many participants acknowledged that the long battery life, helped mitigate some of these concerns. This feature reduced the frequency of recharging, making the device more manageable for users who could access power when they needed to recharge the device.

The main challenge is when there is a distance to where you can access power to recharge the gadget…, once recharged it takes maybe up to three months before the next recharge. (Participant 1)

Another challenge mentioned was the requirement to pair the RTMM with a mobile phone to receive SMS reminders. This was particularly burdensome for participants who either did not own a personal mobile phone or could not afford one.

The way I look at it…, it’s helpful indeed but…, but, since they must work like a pair with a phone. So, possibly for those that are poor, they may not be able to get a phone. (Participant 6)

#### Ethicality

Participants generally had a positive perception toward the intervention in terms of autonomy, and respect to privacy. Participants explained that the discreet design of the RTMM was an important feature as it could easily disguise what is held inside or its intended use as it did not look like a conventional medication pillbox.

And even if I may carry this gadget with me as I am walking on the road people may not be able to tell that there are pills inside. (Participant 10)

However, even though some participants who opted for the alarm appreciated its role in reminding them to take their medication on time, some felt that the alarm put them at risk of unintended disclosure and later decided to have the option disabled.

Yes, as for me, it’s the alarm, yes, it does alert us but if you take that alarm and go somewhere and it sets off right there, it may attract the attention of the people and curiosity to see that which is beeping. (Participant 2)

In terms of the SMS reminders, participants felt that the neutral phrasing of the SMS messages secured their privacy and did not put them at risk of unintended disclosure as the messages did not contain content that explicitly reminded them to take their medication or alluded to their HIV status.

In that case you will be the only one who will know the message because that message may not be easy to understand its meaning even if the message may come while the phone is in someone’s hands even if they may see it, they won’t be able to understand it. (Participant 5)

However, for participants that did not have access to electricity, charging the device in places outside their homes put them at risk of unintended disclosure due to unwanted attention or curiosity from friends or acquaintances as the device would attract attention.

Aah! No. But the usual problem I experience is when I take it to recharge other people get curious like I said. Because that person asked me about it. So, they may get curious and say, let me see what’s inside, so they may end up opening it and discover that there are drugs inside and that can be risky. (Participant 2)

#### Intervention Coherence

Participants generally had a good intervention coherence and understood how the intervention worked and its role in improving their medication adherence.

But from the way we were oriented to the operation of the gadget by ED (Research Assistant), he taught us in a way that we can use the gadget without any difficulties since previously we’d get our drugs from the bottles but now we’re using this gadget with the view that as a country they should observe how people are adhering to their treatment. (Participant 5)

Furthermore, participants understood the interconnection between the RTMM, the reminder SMSs, and the customised adherence feedback and how the different components of the intervention worked together to assist participants adhere to their ART.

When they gave us the gadget, they told us that it was supposed to help us remember to take our medication but also that whenever we take the drugs, there is a way in which it is connected to the computer here at the clinic so that they are also able to monitor our performance on treatment adherence. (Participant 4)

#### Opportunity Costs

In terms of opportunity costs, most of the participants reported no opportunity costs due to their participation in the intervention. However, some participants reported extra clinic visits for customised adherence counselling, disturbances in normal routines, and extra vigilance with privacy as the main opportunity costs.

Concerning extra clinic visits for customised adherence counselling, participants expressed concerns with the frequency and length of clinic visits necessitated by adherence counselling sessions.

Should I be making that trip again?!…. It’s because it’s lengthy and repetitive. (Participant 6)

Furthermore, other participants explained that participating in the intervention disrupted their daily activities.

So, sometimes seven o’clock would strike while I was away in the field as a result I could miss my dosage. I could receive the initial alert. Sometimes I could say possibly I just need to knock off and go home but sometimes people could question that aah he took the trouble to come all the way just to do this bit? (Participant 2)

Privacy concerns also contributed to the perceived opportunity costs. For some participants, ensuring the confidentiality of intervention-related messages required extra vigilance in ensuring that messages are not viewed by anyone else.

Like…, okay, from my perspective there is not any risk because when the message comes, and I’ve seen it I simply go to take the medication and delete the message. (Participant 2)

I just make sure that the phone should never be left unattended or to be left in the hands of someone who doesn’t know that I’m on treatment. (Participant 4)

#### Perceived Effectiveness

Participants felt that participating in the intervention helped them consistently adhere to their drug uptake and schedules. One participant even alluded to the design of the pillbox and the act of storing medication in the pillbox encouraged them to adhere to their medication.

One component that is working well is the one in which we put the drugs in the compartment in the gadget. It’s because there is no room for me to forget to open the compartment on daily basis. (Participant 1)

Many participants reported difficulties adhering to medication before the intervention mainly due to forgetfulness, however, since enrolling into the intervention, their medication adherence improved because the SMS alerts and the audible alarms, for those who opted in, consistently alerted them about their ART intake schedules.

There is a difference, because before we started using the gadget, let’s say when you go visiting (a friend or relative) and the time is up, it was difficult to remember and go back home to take the drugs but here they gave us the gadget and SIM cards which we insert in our phones so, when time is up they alert us that time is up and that we should go and take our medication. It has benefited me a lot because before that thing (the RTMM), it was difficult because I lacked the motivation to take my medication. (Participant 4)

At daybreak in the morning the gadget sends the alarm, and I know that it’s time for medication so it’s difficult that I may forget. The gadget works much better (in improving my adherence) than the time before I started using this gadget. (Participant 7)

I sometimes get the message: ‘It’s about time!’ It’s because it is exactly on time, I can’t skip the time I scheduled to take my medication. I am always alerted to say that I must do this and that. There is a difference because these days I am able to stick to the exact schedule. (Participant 3)

#### Self-efficacy

Many participants were comfortable and confident in their ability to use and participate in the intervention. Participants were particularly confident with how easy it was to load and dispense medication using the device and were impressed with its portability.

One component that is working well is the one in which we put the drugs in the compartment in the gadget. I simply need to open it and take the medication. (Participant 1)

Carrying the gadget when travelling? It is easy because I can even carry it in my bag. (Participant 7)

However, some participants expressed concern on SMSs being sent even after taking medication on time.

Okay, I’ve already said that I can take the medication maybe at 7 am but I then discover that they still send me another reminder at 8 am. Maybe the initial message may arrive at half past six, yes. Then when 7 o’clock strikes I take my medication. At 8 O’clock they still send me another message. (Participant 2)

Furthermore, even though participants were confident in understanding the contents of their adherence graphs or reports at the clinic, some expressed a desire to access the dashboard and graphs from the comfort of their homes using their personal devices rather than visiting the clinic on scheduled days.

Okay, what it means is that it helps you, for example if you’re not adherent to the treatment schedule, that picture (adherence graph) assists you to decide that next time I go to the clinic to meet the clinician, and should he tell me that I never adhered to the drug schedule. Maybe I might have skipped let’s say for four days or else up to five days. Because that graph shows, telling you that on these days you skipped your medication and on the following days you were adherent, and things were okay. (Participant 7)

Okay, I’d say it is okay from the clinic side, but it could be much better if one would be given a chance to be able to see the graph right at home. This could be useful because many people feel shy to go to the clinic so if they could access it at home they would be able to realise that I think I’m doing very well especially for men because find it difficult to go to the hospital due to shyness but if they could access it right at home they would be able to make decisions on time. (Participant 11)

## Discussion

### Key Findings

Our study explored the acceptability of a RTMM-DAT intervention in non-adherent YPLHIV on ART in Malawi enrolled in a randomised controlled trial to investigate the effectiveness of the intervention. Using the TFA, we identified multiple dimensions influencing the interventions acceptability. Overall, the findings demonstrated that the RTMM-DAT was a generally acceptable intervention, with various strengths and challenges identified in the TFA domains. Our general findings are consistent with other studies on the acceptability of RTMM-DATs in PLHIV including YPLHIV and in patients with Tuberculosis (TB), which found these interventions to be generally acceptable among patients and their caregivers [23, 28, 32, 33, 38, 39].

Furthermore, before and after engaging with the intervention, we found that participants faced multiple and complex challenges to ART adherence, such as food insecurity, drug side effects, forgetfulness, and social stigma, consistent with other literature exploring barriers to ART adherence in YPLHIV [7, 40]. These barriers often led to missed doses and, for some, extended periods of poor adherence. Therefore, these underlying and contextual factors underscore the need for developing digital health interventions that not only provide medication intake reminders and customised feedback, but are complemented with interventions that address broader and more complex social-cultural, psychological and economic challenges that negatively impact ART adherence in YPLHIV [41].

### Positive Attitudes and Intervention Self-efficacy

Our study found that participants generally had positive feelings toward the intervention, appreciating the contents of the SMSs, the compact and discreet design of the RTMM, and the customised adherence feedback sessions, which they felt reinforced good adherence behaviours. This aligns with studies in other similar contexts which showed that participants using RTMM-DATs generally had positive feelings towards the intervention [23, 32, 38].

Another important finding was the participants’ high self-efficacy in engaging with the intervention. Their confidence in engaging with all aspects of the intervention is an important feature because RTMM-DATs and similar digital health interventions can only be successfully and effectively deployed at scale in HIV care & treatment clinics if they are easy and intuitive to use and engage with by both the participants and caregivers.

### Intervention Burden and Challenges

Despite positive attitudes, participants identified certain burdens related to the intervention, notably the need for periodic recharging of the RTMM device and the requirement of a personal functional mobile phone to receive reminders. Despite the RTMMs rated battery life of up to 6 months, limited access to electricity at home for some participants was a barrier to consistent device use, consistent with findings in other low-resource settings on similar interventions [32].

Therefore, to mitigate such challenges, in addition to innovating on prolonging the battery life of the RTMMs to address electricity constraints, future implementations of RTMM-DATs in similar contexts should consider prioritising renewable and portable energy solutions, such as solar chargers or swappable and removable batteries to ensure that battery recharging is more convenient for participants with limited access to electricity [32, 38].

### Perceived Effectiveness and Ethicality: Managing Stigma and Privacy

The intervention was viewed as effective in supporting adherence, with participants reporting an improvement in their consistency and motivation to take their ART consistently. However, there were ethicality concerns, particularly the risk of unintended HIV disclosure due to audible alarms or curiosity from others about the RTMM. These concerns underscore the importance of prioritising privacy when designing similar interventions. Therefore, we suggest that future iterations of RTMM-DATs, particularly the smart pillbox, should allow users to enable and disable the alarm at their own convenience rather than by the healthcare provider as is currently the case.

However, despite concerns with the audible alarms, the participants appreciated the neutral SMS phrasing, which safeguarded their privacy as opposed to SMSs that would explicitly inform the participant to remember to take their medication, which could potentially have revealed their HIV status. This is consistent with findings from similar settings like Tanzania in which participants preferred SMSs reminders that were neutral and unrelated to their HIV status [32].

### The Role of the RTMM-DAT in Addressing Forgetfulness and Routine Disruptions

A notable strength of the intervention was its perceived usefulness to participants in addressing forgetfulness, a common barrier to ART adherence [27] through timely SMS reminders and the visual cue of the pillbox itself. The participants’ positive response to the SMS reminders and customised adherence feedback suggests that integrating similar digital adherence support tools in ART programs could be an acceptable intervention to reduce unintentional nonadherence in YPLHIV. However, it should be noted that forgetfulness is usually an indicator of deeper psychological or structural issues that have a negative impact on adherence [42]. Therefore, more comprehensive approaches that address the root cause of forgetfulness in addition to digital adherence support tools could be more effective than implementing reminder systems on their own.

### Implications for Future Research and Practice

There is mixed evidence on the effectiveness of RTMM-DATs in improving ART adherence and viral suppression PLHIV, more especially in YPLHIV [21, 22]. Therefore, even though multiple studies have shown high acceptability of these interventions in PLHIV [23, 28, 38], their mixed performance in improving clinical outcomes reveals that there are more complex sociocultural and psychological factors that moderate the efficacy of such interventions. These moderating factors are especially pronounced in YPLHIV, who must navigate HIV care amidst the physical, social, and economic transitions of adolescence and early adulthood [7].

**Fig. 1:**
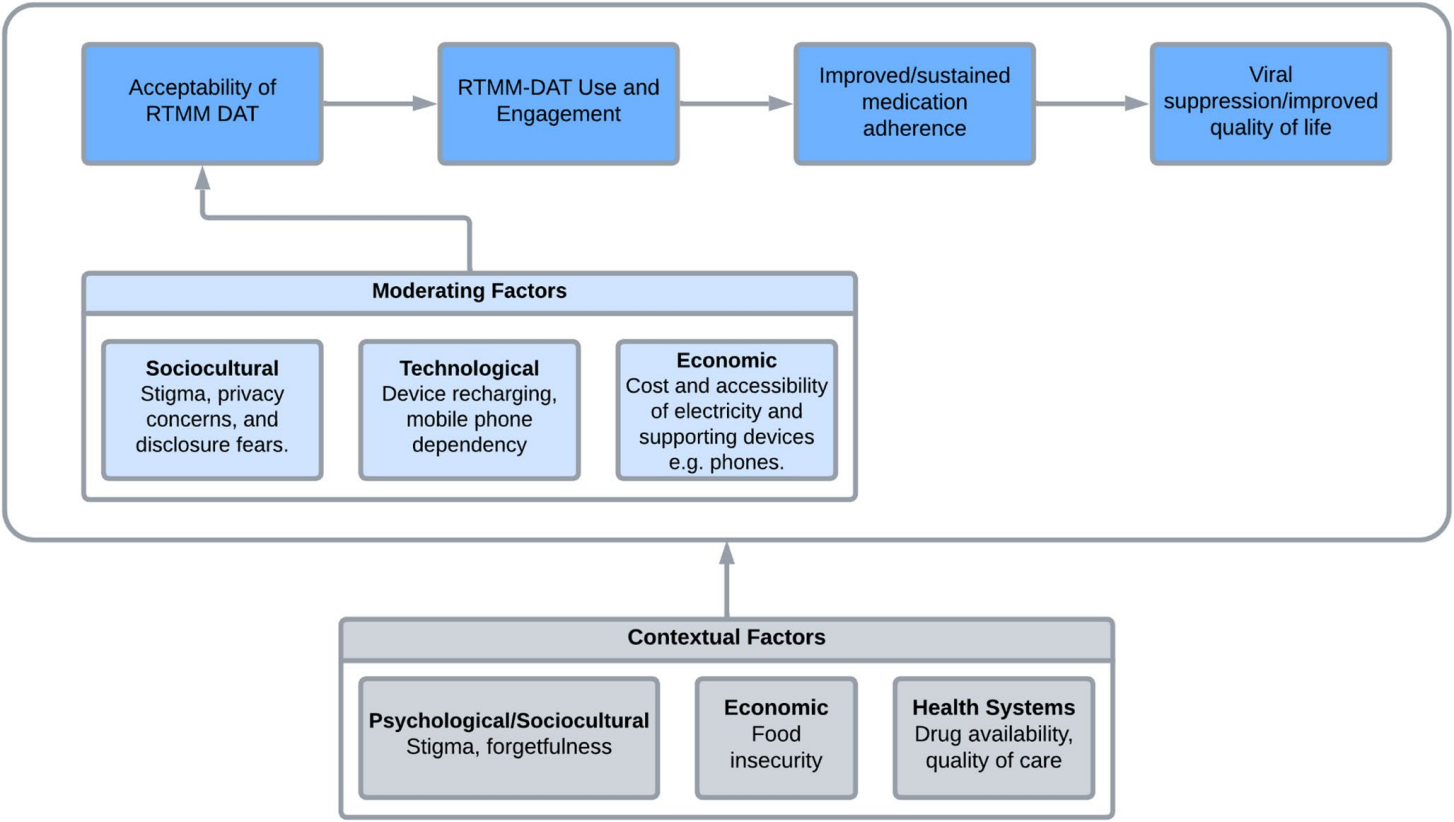
Conceptual framework of RTMM-DAT acceptability and improved clinical outcomes moderated by various contextual factors

Therefore, our findings underscore the importance of tailoring RTMM-DATs to the socio-cultural context of YPLHIV, particularly in low-resource settings like Malawi. RTMM-DATs in HIV/AIDS care and treatment are complex health interventions as they involve multiple interacting components, which can vary in terms of their behaviour, implementation, and context [43, 44]. Therefore, the design, implementation, and evaluation of RTMM-DATs and similar interventions should take into consideration interacting and dynamic contextual and intervention related factors. The acceptability of RTMM-DATs is influenced by several moderators, working within a broader pre-existing system of contextual and pre-existing factors that affect ART adherence in YPLHIV as can be seen in Fig. 1.

Consequently, we propose that future research should focus on optimising these tools for increased accessibility and acceptability, addressing barriers such as phone ownership and charging needs. Additionally, our study highlights the potential benefits of expanding the intervention to allow users to view their adherence data remotely and on their personal devices rather than having to go visit the clinic, potentially enhancing self-management and care, putting the user at the centre of the intervention. Furthermore, providing more user-customisable options like enabling and disabling the alarm at any time of their choice could provide users with more options, control and flexibility, and most importantly, eliminate the risk of unintended disclosure for those who opt for the alarm or find it helpful in certain situations. Therefore, these adjustments could improve the acceptability of the intervention and potentially efficacy; by providing more autonomy and options to YPLHIV using the intervention.

Lastly, the advent of long-acting antiretroviral therapy (LAART) like injectable cabotegravir and rilpivirine poses important questions on the future role of RTMM-DATs and similar digital health interventions in HIV care and treatment. LAART provides new options for YPLHIV by shifting away from the paradigm of daily oral pills, providing more convenient and effective options for YPLHIV who often face adherence challenges and treatment fatigue [45, 46]. In light of this, DATs could evolve beyond supporting adherence to ART into providing treatment support for comorbidities like tuberculosis and non-communicable diseases in PLHIV [33, 47, 48]. Furthermore, they may be used in supporting adherence in PLHIV on a combination of LAART and conventional pill based ART, and also YPLHIV not eligible for LAART [46]. Therefore, RTMM-DATs will still have an important role to play in HIV care and treatment.

### Strengths and Limitations

This study has several limitations. First, participants were drawn from a clinical trial setting, which may not fully represent the behaviour YPLHIV outside of structured research environments due to the Hawthorne effect [49]. Second, as our analysis on the acceptability of the intervention was confined to the TFA domains, some important aspects of the participants’ experiences outside the theoretical framework may not have been fully captured. Third, even though YPLHIV were considered a relatively homogenous group, they are at different stages of their life, and the factors that affect the acceptability could be significantly vary. Therefore, future studies with larger, more diverse samples across varied age ranges and settings would provide more granular insights into the real-world acceptability and applicability of RTMM-DATs in HIV care and treatment.

However, our study had several strengths. First, we interviewed participants who were concurrently enrolled in the intervention, allowing them to share their experiences as they occurred. Second, in addition to collecting data on the acceptability of the intervention, we explored barriers to ART adherence participants faced or were currently facing. This allowed us to define the context in which the intervention was implemented, grounding our conclusions to the real-world context.

## Conclusions

In conclusion, the RTMM-DAT is a promising tool for improving ART adherence in YPLHIV, with generally favourable acceptability. By addressing technological and socio-cultural barriers, RTMM-DATs in combination with other psychosocial and biological interventions may be pivotal in supporting ART adherence in YPLHIV, potentially improving treatment outcomes and quality of life in this demographic.

## Supplementary Information

Below is the link to the electronic supplementary material.

Supplementary material 1 (DOCX 17.8 kb)

## Data Availability

The data supporting the findings of this study are qualitative transcripts. Due to the sensitive nature of the data and to protect participant confidentiality, the full transcripts are not publicly available. However, anonymized excerpts relevant to specific research questions may be made available upon reasonable request to the corresponding author, subject to ethical clearance from the COMREC.
