# Supplementary material for: The Acceptability of a Real-Time Medication Monitoring-Based Digital Adherence Tool Among Young People Living with HIV in Malawi
Source: AIDS Behav. 2025 Sep 23;30(2):379–91. doi: 10.1007/s10461-025-04885-7 (PMC12929300; doi:10.1007/s10461-025-04885-7)
Supplement: Supplementary file 1 — Supplementary material 1 (DOCX 17.8 kb) [file 10461_2025_4885_MOESM1_ESM.docx]

**QUESTIONNAIRE**

Marital Status:_______________________

Religion:______________________

How did you realize that you are HIV positive?

**Situation at home**

- Where do you live? Who do you live with?
- Are both parents alive?
- What type of house do you live in? (iron-sheet roofed/ grass thatched/electricity/water?)
- Do you always have enough food to eat at home? Do they ever have problems finding daily food at home?

**Examining experiences with HIV care and treatment**

When and how did you know you had HIV?

What has been your experience with HIV care and treatment

- When did you start taking your ART medications?
- Who is main guardian?
- Experience accessing care and treatment services?

Why is adhering to medication important for someone living with HIV?

What strategies do you use to make sure that you do not forget to take your medication?

What challenges have you been facing in adhering to your medication according to clinic guidance?

Have you taken any steps initiated by yourself or a health care worker to help improve your medication adherence?

**Examining perceptions and attitudes towards the intervention**

What are your views towards the digital adherence tool you are currently using? Probe about their views and experience with the following

- Probe about their view on the RTMM device
- triggered reminders
- customized adherence feedback

How do you think using the gadget, reminder SMSs, and adherence feedback can help you to take medication according to schedule?

How useful do you think the digital adherence tool was to you? (did it help the participants' adherence). Probe about what aspects about the intervention was most useful to them

- Device
- Triggered reminders
- Adherence feedback

What aspects of the intervention did they like? Probe about

- RTMM device
- Triggered reminders
- Customized adherence feedback

What aspects of the intervention did they not like? Probe about

- RTMM device
- Triggered reminders
- Customized adherence feedback

What risks were associated with the use of the device? Probe about

- Unwanted attention
- Unintended disclosure
- Physical abuse
- Verbal abuse
- Stigma
- Loss of privacy/being watched
- Loss of autonomy
- Loss of friends/acquaintances/intimate partners
- Change in behaviour patterns/personal relationships

What kind of challenges are you experiencing from using the electronic device?

- Probe on technical challenges: use of the device, access to power, portability, receiving reminder SMSs, network issues
- Probe about whether the intervention has added demand on time, effort, space

Who do you think should be prioritized in using the electronic device? Why do you think so?

What aspects of the intervention can be improved? Probe about what areas of the intervention can be improved, adapted, or entirely removed.

Would you recommend the use of the intervention (device) to others?

Any additional comments?
